# Supplementary material for: Mechanisms governing the pioneering and redistribution capabilities of the non-classical pioneer PU.1
Source: Nat Commun. 2020 Jan 21;11:402. doi: 10.1038/s41467-019-13960-2 (PMC6972792; doi:10.1038/s41467-019-13960-2)
Supplement: Supplementary file 7 — Source data [file 41467_2019_13960_MOESM7_ESM.zip › Source_Data/FigureS5/FigureS5A_MotifsCommonPeaks/homerResults/motif27.similar.html]

motif27

## Information for motif27

A
T
G
C
C
A
G
T
A
C
G
T
T
A
G
C
A
G
T
C
C
A
G
T
T
A
G
C
A
G
T
C
A
G
C
T
T
G
A
C
A
G
T
C
G
A
C
T
T
A
G
C
  
Reverse Opposite:  

A
T
C
G
C
T
G
A
A
C
T
G
A
C
T
G
T
C
G
A
A
C
T
G
A
T
C
G
G
T
C
A
A
C
T
G
A
T
C
G
T
G
C
A
G
T
C
A
A
T
C
G
  

|  |  |
| --- | --- |
| p-value: | 1e-55 |
| log p-value: | -1.270e+02 |
| Information Content per bp: | 1.746 |
| Number of Target Sequences with motif | 356.0 |
| Percentage of Target Sequences with motif | 4.01% |
| Number of Background Sequences with motif | 485.9 |
| Percentage of Background Sequences with motif | 1.28% |
| Average Position of motif in Targets | 95.0 +/- 51.5bp |
| Average Position of motif in Background | 98.2 +/- 76.0bp |
| Strand Bias (log2 ratio + to - strand density) | 0.0 |
| Multiplicity (# of sites on avg that occur together) | 1.00 |
| Motif File: | file (matrix) reverse opposite |

### Similar de novo motifs found

|  |  |  |  |  |  |  |  |
| --- | --- | --- | --- | --- | --- | --- | --- |
| Rank | Match Score | Redundant Motif | P-value | log P-value | % of Targets | % of Background | Motif file |
| 1 | 0.781 | C A G T A T G C G T A C C A G T A G T C G T A C G C A T A G T C G T A C G C A T A G T C C G A T A C G T T A G C | 1e-29 | -67.747773 | 4.29% | 2.05% | motif file (matrix) |
| 2 | 0.882 | T A G C A T G C A G C T A C T G A G T C A G T C G A T C A G T C A G T C G C A T A G T C A G T C G A T C T G A C | 1e-13 | -30.345660 | 1.51% | 0.65% | motif file (matrix) |
